# Supplementary material for: Assessment of Micro- and Nanoplastic Composition (Polymers and Additives) in the Gastrointestinal Tracts of Ebro River Fishes
Source: Molecules. 2022 Dec 28;28(1):239. doi: 10.3390/molecules28010239 (PMC9821878; doi:10.3390/molecules28010239)
Supplement: Supplementary file 1 [file molecules-28-00239-s001.zip › molecules-2077067-supplementary.pdf]

Supplementary material

ASSESSMENT OF MICRO- AND NANOPLASTICS COMPOSITION (POLYMERS AND ADDITIVES) IN THE GASTROINTESTINAL TRACT OF THE EBRO RIVER FISH

Maria Garcia-Torné[1], Esteban Abad[1], David Almeida[2,3], Marta Llorca[1,*], Marinella Farré[1,*]

[1] Institute of Environmental Assessment and Water Research (IDAEA-CSIC), C/ Jordi Girona, 18-26, 08034, Barcelona (Spain).

[2] GRECO, Institute of Aquatic Ecology, University of Girona, Campus Montilivi, 17003, Girona (Spain).

[3] Department of Basic Medical Sciences, School of Medicine, Universidad San Pablo-CEU, CEU Universities, Urbanización Montepríncipe, 28668, Boadilla del Monte (Spain).

***Corresponding authors:**

mfuqam@cid.csic.es

mlcqam@cid.csic.es

Table S1. Visual observations and particle recount.

Species	Samples	Black fibres	Red fibres	Green fibres	White fibres	Black spots	Fragments/Comments
	Control				9	288	Brownish spots
Dicentrarchus labrax	**DLT1**		1			3458*	*Irregular black spots
	**DLX1**						36 irregular brownish spots
	**DLX2**		2		36		3 irregular brownish spots
Pseudorasbora parva	**PPT1**		1	7			2 Green spots, 2 crystals
Silurus glanis	**SGT1**		1			720	
Alburnus alburnus	**AAX1**		1				
	**AAX5**						
	**AAX6**	2	24				
	**AAX7**	14	1	2	3		
	**AAX8**		1				
	**AAX9**	86	24	36	36	24	
Barbus graellsii	**BAT1**		3	2		13	
Carassius auratus	**CAT1**	2	2				
	**CAT2**		1	4			
	**CAT3**					144*	*Irregulars
	**CAT4**		5	2		108	
	**CAX1**			3		36*	*Brackish/brownish
	**CAX2**		1	1			
Ictalurus punctatus	**ICT1**		2				Irregular black and brown spots
	**ICT2**		2	1			Irregular black and brown spots
Rutilus rutilus	**RRX1**	230	48	48	386		
	**RRX2**	62	3				
Squalius laietanus	**SLT1**						
	**SLT2**		2	6		264*	*Full of spots
	**SLX1**					48	
	**SLX2**		3	2		24*	*Irregulars
	**SLX3**						
Cyprinus carpio	**CYT1**		2	1			Irregular black and brown spots
	**CYX1**				4		Brownish spots
Liza sp.	**LIT1**		3	2			Brown spots
	**LIT2**					228	
	**LIT3**		2			108	
	**LIT4**		2	1			
	**LIX1**		1		36		2 bright spots
	**LIX2**		1		3		
Mugil cephalus	**MCT1**			2		108	Crystals
	**MCT2**					120	
	**MCT3**		2	2			
	**MCX1**				5		192 blackish/brownish spots
	**MCX1rep**	22	3			72	

	Carnivores
	Omnivores
	Detritivores

Table S2. Compounds' parameters used for the prioritisation.

Compounds	Formula	Detection frequency (%)	Half-life (biodegradation)	log BAF	Toxicity	
Isopropyl myristate	C17 H34 O2	21.951	5.69 days	2.351	LD50(rat-oral) >	16 mL/kg
2,5-di-tert-Butylhydroquinone	C14 H22 O2	21.951	20.6 days	2.037		
2,6-di-tert-butyl-4-ethylphenol	C16 H26 O	14.634	14.3 days	3.620		
2,6-Dicyclohexylphenol	C18 H26 O	2.439	46.2 days	2.759		
Citroflex A-4	C20 H34 O8	51.220	5.67 days	1.100		
1,2-Benzisothiazolin-3-one	C7 H5 N O S	17.073	3.74 days	0.118	LD50(rat-oral) =	1020 mg/kg
2,5-Bis(tert-butylperoxy)-2,5-dimethylhexane	C16 H34 O4	17.073	20.4 days	3.905	LD50(rat-oral) >	3200 mg/kg
Dodecamethylpentasiloxane	C12 H36 O4 Si5	9.756	215 days	5.912		
Oil Red O	C26 H24 N4 O	4.878	47.16 days	4.208		
2-Ethylpentyl 3-[4-hydroxy-3,5-bis(2-methyl-2-propanyl)phenyl]propanoate	C24 H40 O3	2.439	0.88 days	1.585		
5-methyl-2,2'-(1,2-Ethenediyldi-4,1-phenylene)-Bisbenzoxazole	C29 H20 N2 O2	2.439	25.9 days	6.716		
Di-n-Amyl phthalate	C18 H26 O4	2.439	4.78 days	1.871		
Bis(2-ethylhexyl) phthalate	C24 H38 O4	9.756	4.81 days	3.017	LD50(rat-oral) =	30000 mg/kg
bisphenol A diglycidyl ether	C21 H24 O4	2.439	5.75 days	2.603	LD50(rat-oral) >	1000 mg/kg
Tetrahydrofurfuryl acrylate	C8 H12 O3	19.512	4.64 days	0.216		
N,N-Bis(2-hydroxyethyl)dodecanamide	C16 H33 N O3	24.390	5.8 days	1.064	LD50(rat-oral) =	2700 mg/kg
Dicyclohexyl phthalate	C20 H26 O4	2.439	4.08 days	2.136	LD50(rat-oral) >	3200 mg/kg
12-HSA	C18 H36 O3	14.634	7.28 days	3.129		
Laurolactam	C12 H23 N O	36.585	5.92 days	1.631		
Diisobutyl adipate	C14 H26 O4	43.902	4.6 days	1.099		
1,1-bis(tert-butylperoxy)-3,3,5-trimethylcyclohexane	C17 H34 O4	12.195	9.2 days	4.469	LD50(rat-oral) =	12918 mg/kg
2,2'-Biquinoline	C18 H12 N2	2.439	105 days	2.560		

Compounds	Formula	Detection frequency (%)	Half-life (biodegradation)	log BAF	Toxicity	
Acridine	C13 H9 N	2.439	30.3 days	2.076	LD50(rat-oral) =	2000 mg/kg
3-(3,5-di-tert-butyl-4-hydroxyphenyl)propanoic acid	C17 H26 O3	7.317	5.86 days	2.565		
2-Ethylhexyldiphenyl phosphate	C20 H27 O4 P	4.878	4.23 days	2.436	LD50(rat-oral) =	7500 mg/kg
Oleic acid	C18 H34 O2	9.756	5.4 days	5.179	LD50(rat-oral) =	25000 mg/kg
2,4-Di-tert-butylphenyl 3,5-di-tert-butyl-4-hydroxybenzoate	C29 H42 O3	2.439	22.6 days	3.459		
2,6-di-tert-Butylphenol	C14 H22 O	12.195	14.8 days	2.806		
dehydroabietic acid	C20 H28 O2	4.878	5.69 days	3.261	LD50(rat-oral) =	1710 mg/kg
1,3-Diisopropylbenzene	C12 H18	9.756	10.2 days	3.113	LD50(rat-oral) =	7400 mg/kg
2-tert-Butyl-4-methoxyphenol	C11 H16 O2	9.756	3.7 days	2.041	LD50(rat-oral) =	2910 mg/kg
4-Nonylphenol	C15 H24 O	2.439	3.73 days	2.349	LD50(rat-oral) =	1620 mg/kg
Trihexyl 1,2,4-benzenetricarboxylate	C27 H42 O6	2.439	5.69 days	0.165	LD50(rat-oral) =	5.66 mL/kg
Dodecanedioic acid	C12 H22 O4	31.707	8 days	2.012		
3,3,5-Trimethylcyclohexyl methacrylate	C13 H22 O2	2.439	4.47 days	2.448		
Decanoic acid	C10 H20 O2	17.073	4.99 days	2.597	LD50(rat-oral) =	3320 mg/kg
Hexamethyldisiloxane	C6 H18 O Si2	17.073	64.8 days	3.895	LD50(rat-oral) >	5000 mg/kg
3-Vinyl-7-oxabicyclo[4.1.0]heptane	C8 H12 O	36.585	3.38 days	1.118	LD50(rat-oral) =	2 mL/kg
Nonanoic acid	C9 H18 O2	17.073	5 days	2.197	LD50(rat-oral) >	5000 mg/kg
Bis(2,2,6,6-tetramethyl-4-piperidyl)sebacate	C28 H52 N2 O4	4.878	14.5 days	2.334		
Dimethyl sebacate	C12 H22 O4	29.268	3.68 days	0.809		
Azelaic acid	C9 H16 O4	48.780	7.5 days	0.642	LD50(rat-oral) >	5000 mg/kg
Bis(8-methylnonyl) sebacate	C30 H58 O4	2.439	8.46 days	0.017		
Dibutyl sebacate	C18 H34 O4	2.439	5.68 days	1.459	LD50(rat-oral) =	14870 mg/kg
Pentaerythritol tetravalerate	C25 H44 O8	2.439	5.66 days	0.801		
Stearamide	C18 H37 N O	2.439	17.4 days	2.232		

Compounds	Formula	Detection frequency (%)	Half-life (biodegradation)	log BAF	Toxicity	
Mono(2-ethylhexyl) phthalate (MEHP)	C16 H22 O4	17.073	3.53 days	2.150		
N-Caprylyldiethanolamine	C12 H27 N O2	17.073	5.75 days	0.477		
exo-1,7,7-Trimethylbicyclo(2.2.1)hept-2-yl acrylate	C13 H20 O2	9.756	3.56 days	2.414		
Diisobutyl azelate	C17 H32 O4	2.439	5.68 days	1.335		
Ethyl oleate	C20 H38 O2	2.439	4.03 days	2.840	LD50(rat-oral) >	5000 mg/kg
Trimethylolpropane trimethacrylate	C18 H26 O6	7.317	5.67 days	1.100		
2-Ethoxyethyl acrylate	C7 H12 O3	21.951	4.3 days	0.096	LD50(rat-oral) =	1070 mg/kg
2,6-di-tert-Butyl-4-methoxyphenol	C15 H24 O2	12.195	6.1 days	2.526		
2,5-Bis(2-methyl-2-butanyl)-1,4-benzenediol	C16 H26 O2	14.634	7.47 days	2.270	LD50(rat-oral) =	2000 mg/kg
Dibutyl itaconate	C13 H22 O4	2.439	4.63 days	1.054		
Dicyclohexylamine	C12 H23 N	2.439	34.7 days	3.074	LD50(rat-oral) =	373 mg/kg
Oleonitrile	C18 H33 N	12.195	5.6 days	4.695	LD50(rat-oral) >	10000 mg/kg
Sebacic acid	C10 H18 O4	29.268	7.56 days	1.179	LD50(rat-oral) =	14375 mg/kg
Bisphenol AF	C15 H10 F6 O2	2.439	1.93 days	2.808	LD50(rat-oral) =	3400 mg/kg
o-tert-Octylphenol	C14 H22 O	2.439	3.1 days	2.976	LD50(rat-oral) >	2000 mg/kg
2-Diethylaminoethyl methacrylate	C10 H19 N O2	26.829	4.29 days	0.450	LD50(rat-oral) =	4696 mg/kg
Decyl octyl phthalate	C26 H42 O4	4.878	8.8 days	0.063	LD50(rat-oral) =	45000 mg/kg
BIS T-BUTYLDIOXYISOPROPYLBENZENE	C20 H34 O4	2.439	55.57 days	6.153		
Tributyl phosphate	C12 H27 O4 P	31.707	3.68 days	1.843	LD50(rat-intraperitoneal) =	251.2 mg/kg
2-Ethylhexanoic acid	C8 H16 O2	19.512	4.78 days	1.579	LD50(rat-oral) =	1600-3000 mg/kg
Butyldiglycol acetate	C10 H20 O4	19.512	3.68 days	0.219	LD50(rat-oral) =	6500 mg/kg
Benzophenone	C13 H10 O	4.878	5.25 days	1.379	LD50(rat-oral) >	10000 mg/kg
2-Mercaptobenzothiazole	C7 H5 N S2	9.756	13 days	0.765	LD50(rat-oral) =	20227 mg/kg

Compounds	Formula	Detection frequency (%)	Half-life (biodegradation)	log BAF	Toxicity	
vinyl propanoate	C5 H8 O2	12.195	4.13 days	0.230	LD50(rat-oral) =	4760 mg/kg
Dibutyl hexanedioate	C14 H26 O4	0.268	5.7 days	1.100	LD50(rat-oral) =	12900 mg/kg
2-Butoxyethanol acetate	C8 H16 O3	9.756	3.81 days	0.410	LD50(rat-oral) =	2400 mg/kg
12-Hydroxy-9-octadecenoic acid	C18 H34 O3	14.634	3.31 days	3.259		
Diphenolic acid	C17 H18 O4	2.439	6.28 days	1.926		
Tributylamine	C12 H27 N	4.878	3.9 days	2.233	LD50(rat-oral) =	4.92 mL/kg
Tetramethylurea	C5 H12 N2 O	14.634	7.6 days	-0.029	LD50(rat-oral) =	114 mg/kg
DIISOOCTYL DITHIOPHOSPHATE	C16 H35 O2 P S2	2.439	7.06 days	3.907	LD50(rat-oral) =	3100 mg/kg
4-Toluic acid	C8 H8 O2	9.756	3.72 days	1.241	LD50(rat-intraperitoneal) =	874 mg/kg
Bis(3-methoxypropyl) adipate	C14 H26 O6	7.317	3.8 days	0.280		
Diethyl sebacate	C14 H26 O4	7.317	5.67 days	1.100	LD50(rat-oral) =	14470 mg/kg
3,3-Dimethyl-1,5-dioxacyclopentadecane-6,15-dione	C15 H26 O4	4.878	5.7 days	1.454		
4-tert-Amylphenol	C11 H16 O	4.878	3.73 days	2.362	LD50(rat-oral) =	1830 mg/kg
Citroflex 4	C18 H32 O7	4.878	5.67 days	0.805		
3-Vinyltoluene	C9 H10	2.439	1.75days	2.321		
Cyclohexyl methacrylate	C10 H16 O2	2.439	5.2 days	1.630		
6,6',6''-(1,3,5-Triazine-2,4,6-triyltriimino)trihexanoic acid	C21 H36 N6 O6	2.439	5.02 days	1.836		
cetyl sulfate	C16 H34 O4 S	17.073	0.42 days	2.225		
Isophorone	C9 H14 O	17.073	3.7 days	0.601	LD50(rat-oral) =	1000-3450 mg/kg
METHYL OLEATE	C19 H36 O2	4.878	1.96 days	2.699		
Diphenylamine	C12 H11 N	14.634	6.65 days	2.393	LD50(rat-oral) >	5000 mg/kg
12-Aminododecanoic acid	C12 H25 N O2	14.634	7.5 days	0.033		
N-Butylbenzenesulfonamide	C10 H15 N O2 S	14.634	4.3 days	1.217	LD50(rat-oral) =	2050 mg/kg

Compounds	Formula	Detection frequency (%)	Half-life (biodegradation)	log BAF	Toxicity	
Monobutyl phthalate	C12 H14 O4	2.439	3.5 days	1.407		
2-Benzyloxyethanol	C9 H12 O2	7.317	4.45 days	0.066	LD50(rat-oral) =	1190 mg/kg
4-Methylphenol	C7 H8 O	12.195	5.34 days	0.870	LD50(rat-oral) =	270 mg/kg
Triphenylphosphine oxide	C18 H15 O P	4.878	122 days	1.667	LD50(rat-oral) =	3000 mg/kg
(Hydroxyethyl)methacrylate	C6 H10 O3	9.756	4.46 days	-0.012	LD50(rat-oral) =	11200 mg/kg
Oxepanone	C6 H10 O2	9.756	4.98 days	0.024	LD50(rat-oral) =	4290 mg/kg
1,1,3,3-Tetramethyl-1,3-divinyldisiloxane	C8 H18 O Si2	2.439	15.1 days	4.936	LD50(rat-oral) >	10000 mg/kg
1,3:24- Bis (3,4-dimethylobenzylideno) sorbitol (DMDBS)	C24 H30 O6	2.439	0.13 days	1.695		
4-Acetylphenetole	C10 H12 O2	7.317	4.43 days	1.234		
(+/-)-Camphor	C10 H16 O	2.439	5.4 days	1.682	LD50(rat-intraperitoneal) =	956 mg/kg
4-Methylbenzotriazole	C7 H7 N3	2.439	0.19 days	0.742		
5,8,11,13,16,19-Hexaoxatricosane	C17 H36 O6	2.439	4.29 days	0.643	LD50(rat-oral) =	1746 mg/kg
Benzotriazole	C6 H5 N3	2.439	3.99 days	0.546	LD50(rat-oral) =	560 mg/kg
o-Toluidine	C7 H9 N	2.439	3.8 days	0.421	LD50(rat-oral) =	670 mg/kg
Suberic acid	C8 H14 O4	7.317	5.48 days	0.381		
(Triethoxymethoxy)ethane	C9 H20 O4	26.829	5.2 days	0.839		
Undecanedioic acid	C11 H20 O4	14.634	7.94 days	1.613		
Bis(2-butoxyethyl) adipate	C18 H34 O6	14.634	4.23 days	0.805	LD50(rat-intraperitoneal) =	600 mg/kg
Caprolactam	C6 H11 N O	14.634	4.57 days	0.040	LD50(rat-oral) =	1200 mg/kg
(Vinyloxy)cyclohexane	C8 H14 O	12.195	4.21 days	1.624		
Diacetone acrylamide	C9 H15 N O2	7.317	4.8 days	-0.036	LD50(rat-oral) =	1770 mg/kg
Dibutyl malate	C12 H22 O5	7.317	4.64 days	0.277	LD50(rat-oral) >	9699 mg/kg
PEG-4	C8 H18 O5	12.195	7.59 days	-0.049	LD50(rat-oral) =	30000 mg/kg

Compounds	Formula	Detection frequency (%)	Half-life (biodegradation)	log BAF	Toxicity	
Diallyl adipate	C12 H18 O4	36.585	4.62 days	0.788		
benzyl alcohol	C7 H8 O	2.439	6.6 days	0.190		
mequinol	C7 H8 O2	2.439	5.35 days	0.587	LD50(rat-oral) =	1600 mg/kg
Triethylamine	C6 H15 N	2.439	8.84 days	0.430	LD50(rat-oral) =	17000 mg/kg
Triethylene glycol	C6 H14 O4	12.195	7.58 days	-0.049		
Butyl glycolate	C6 H12 O3	4.878	4.64 days	-0.025		
Cyclohexane-1,4-dimethanol	C8 H16 O2	2.439	5.37 days	0.505	LD50(rat-oral) =	3200 mg/kg
Dimethyl phthalate	C10 H10 O4	2.439	3.81 days	0.291	LD50(rat-oral) =	6800 mg/kg
hexahydrophthalic anhydride	C8 H10 O3	2.439	5.32 days	1.187	LD50(rat-oral) =	2270 mg/kg
Benzothiazole	C7 H5 N S	14.634	8.45 days	0.950	LD50(rat-oral) =	380 mg/kg
12-Crown-4	C8 H16 O4	2.439	14.9 days	-0.018	LD50(rat-oral) =	2830 mg/kg
2-Anisidine	C7 H9 N O	2.439	3.58 days	0.337	LD50(rat-oral) =	1800 mg/kg
Butyl 2,3-dihydroxypropyl sebacate	C17 H32 O6	2.439	5.7 days	0.800		
Ethyl terephthalate	C12 H14 O4	2.439	5.13 days	0.889		
PPG n4	C12 H26 O5	14.634	3.68 days	-0.044		
3,6,9,12,15,18-Hexaoxaicosane-1,20-diol	C14 H30 O8	9.756	7.6 days	-0.049		
2,5-Bis(hydroxymethyl)furan	C6 H8 O3	14.634	6.57 days	-0.041		
Bis(2-(2-butoxyethoxy)ethyl) phthalate	C24 H38 O8	2.439	4.23 days	0.992	LD50(rat-oral) =	9700 mg/kg
Bis[4-(vinyloxy)butyl] 1,6-hexanediylbiscarbamate	C20 H36 N2 O6	2.439	0.01 days	0.821		
4,4'-Dithiodimorpholine	C8 H16 N2 O2 S2	2.439	11.6 days	-0.049	LD50(rat-oral) =	4300 mg/kg
Diethylene glycol	C4 H10 O3	4.878	6.13 days	-0.049	LD50(rat-oral) =	12565 mg/kg
2-Decyl-2-(hydroxymethyl)-1,3-propanediol	C14 H30 O3	14.634	0.29 days	1.796		
2-Hydroxybenzothiazole	C7 H5 N O S	14.634	5.54 days	0.719		
Allyl 2,2,3,3-tetrafluoropropyl ether	C6 H8 F4 O	14.634	0.42 days	1.174		

Compounds	Formula	Detection frequency (%)	Half-life (biodegradation)	log BAF	Toxicity	
2,6-Xylidine	C8 H11 N	12.195	11.4 days	0.780	LD50(rat-oral) =	2170 mg/kg
Cyclohexanecarboxylic acid	C7 H12 O2	12.195	5.8 days	0.983	LD50(rat-oral) =	3265 mg/kg
Valeric acid	C5 H10 O2	9.756	4.28 days	0.506	LD50(rat-oral) =	4760 mg/kg
(4-Methoxybutoxy)(trimethyl)silane	C8 H20 O2 Si	7.317	0.71 days	1.525		
1,8-Diazacyclotetradecane-2,7-dione	C12 H22 N2 O2	4.878	0.01 days	0.094		
2,5-Dimethyl-3-hexyne-2,5-diol	C8 H14 O2	9.756	4.6 days	0.132		
1,2,3,4-Tetramethyl-1,3-cyclopentadiene	C9 H14	2.439	7.16 days	3.021		
2,3,6-Trimethylphenol	C9 H12 O	2.439	8.02 days	1.237		
2-mercaptomethylbenzimidazole	C8 H8 N2 S	2.439	0.05 days	0.908	LD50(rat-oral) =	340 mg/kg
Tris(2-chloroethyl) phosphate	C6 H12 Cl3 O4 P	2.439	3.68 days	0.540		
PPG n5	C15 H32 O6	12.195	0.01 days	-0.042		
4-Methyl-2-oxotetrahydro-2H-pyran-4-yl methacrylate	C10 H14 O4	7.317	0.003 days	0.046		
2-(pent-4-ynyl)-2-oxazoline	C8 H11 N O	2.439	0.16 days	1.431		
2-Methoxy-5-methylaniline	C8 H11 N O	2.439	3.49 days	0.710	LD50(rat-oral) =	364 mg/kg
2-Methyl-2-propanyl 3-(2-hydroxyethoxy)propanoate	C9 H18 O4	2.439	0.01 days	-0.018		
3-(3-pyridinyl)propanoic acid	C8 H9 N O2	2.439	0.07 days	0.263		
Cyclohexanecarboxylate	C7 H11 O2	2.439	0.41 days	0.983		
tetramethyl-1,3-disiloxanediol	C4 H14 O3 Si2	7.317	7.6 days	0.794	LD50(rat-oral) =	794 mg/kg

Table S3. List of samples and sampling details. (Species (sp), fork length [1], total length [2], body mass (BM)).

date	code	Fishing order	site	sp	FL (mm)	TL (mm)	BM (g)	Liver weight (g)	plasma	Gut	Liver	Muscle
03/04/2019	BAT1	4	TORTOSA	*Barbus graellsii*	90	100	7.2	-		✔		✔
03/04/2019	CAT1	1	TORTOSA	*Carassius auratus*	89	93	10.9	-		✔		✔
03/04/2019	CAT2	2	TORTOSA	*Carassius auratus*	85	92	12.2	-		✔		✔
03/04/2019	CAT3	6	TORTOSA	*Carassius auratus*	290	320	864	-	✔	✔	✔	✔
03/04/2019	CAT4	11	TORTOSA	*Carassius auratus*	117	125	34	-		✔		✔
03/04/2019	CYT1	20	TORTOSA	*Cyprinus carpio*	580	640	3050	66.1	✔	✔	✔	✔
03/04/2019	DLT1	12	TORTOSA	*Dicentrarchus labrax*	364	386	516.2	7	✔	✔	✔	✔
03/04/2019	ICT1	7	TORTOSA	*Ictalurus punctatus*	590	630	2700	53	✔	✔	✔	✔
03/04/2019	ICT2	9	TORTOSA	*Ictalurus punctatus*	580	620	2140	34.8	✔	✔	✔	✔
03/04/2019	LIT1	3	TORTOSA	*Liza sp.*	428	470	962.8	9.6	✔	✔	✔	✔
03/04/2019	LIT2	13	TORTOSA	*Liza sp.*	440	480	942	9.7	✔	✔	✔	✔
03/04/2019	LIT3	14	TORTOSA	*Liza sp.*	350	380	467	6.8	✔	✔	✔	✔
03/04/2019	LIT4	17	TORTOSA	*Liza sp.*	460	520	1153	13.4	✔	✔	✔	✔
03/04/2019	MCT1	16	TORTOSA	*Mugil cephalus*	520	570	1562	20	✔	✔	✔	✔
03/04/2019	MCT2	18	TORTOSA	*Mugil cephalus*	410	450	1014	10.2	✔		✔	✔
03/04/2019	MCT3	19	TORTOSA	*Mugil cephalus*	530	590	1886	23	✔	✔	✔	✔
03/04/2019	PPT1	8	TORTOSA	*Pseudorasbora parva*	74	80	5.4	-		✔		✔
03/04/2019	SGT1	10	TORTOSA	*Silurus glanis*	-	1000	7600	97	✔	✔	✔	✔
03/04/2019	SLT1	5	TORTOSA	*Squalius laietanus*	94	100	9	-		✔		✔
03/04/2019	SLT2	15	TORTOSA	*Squalius laietanus*	94	99	11	-		✔		✔
02/04/2019	AAX1	2	XERTA	*Alburnus alburnus*	91	97	4.6	-		✔		✔
02/04/2019	AAX5	8	XERTA	*Alburnus alburnus*	85	93	6.9	-		✔		✔
02/04/2019	AAX6	9	XERTA	*Alburnus alburnus*	70	76	3.1	-		✔		✔
02/04/2019	AAX7	10	XERTA	*Alburnus alburnus*	65	69	1.9	-		✔		✔
02/04/2019	AAX8	11	XERTA	*Alburnus alburnus*	85	90	4.7	-		✔		✔
02/04/2019	AAX9	12	XERTA	*Alburnus alburnus*	61	65	2	-		✔		✔
02/04/2019	CAX1	15	XERTA	*Carassius auratus*	129	140	43.5	(< 5)		✔	✔	✔
02/04/2019	CAX2	19	XERTA	*Carassius auratus*	114	123	31.8	-		✔	✔	✔

02/04/2019	CYX1	17	XERTA	*Cyprinus carpio*	650	710	5750	40.4	✓	✓	✓	✓
02/04/2019	DLX1	1	XERTA	*Dicentrarchus labrax*	442	460	952	8.9	✓	✓	✓	✓
02/04/2019	DLX2	16	XERTA	*Dicentrarchus labrax*	580	620	2356	20.7	✓	✓	✓	✓
02/04/2019	LIX1	20	XERTA	*Liza sp.*	360	400	1675	21.9		✓	✓	✓
02/04/2019	LIX2	21	XERTA	*Liza sp.*	360	400	470	?	✓	✓	✓	✓
02/04/2019	MCX1	3	XERTA	*Mugil cephalus*	550	590	1789	23.6	✓	✓	✓	✓
02/04/2019	RRX1	22	XERTA	*Rutilus rutilus*	115	126	17.4	-		✓		✓
02/04/2019	RRX2	23	XERTA	*Rutilus rutilus*	112	122	18.8	-		✓		✓
02/04/2019	SLX1	7	XERTA	*Squalius laietanus*	82	87	6.2	-		✓		✓
02/04/2019	SLX2	13	XERTA	*Squalius laietanus*	83	90	5.8	-		✓		✓
02/04/2019	SLX3	14	XERTA	*Squalius laietanus*	66	72	3.7	-		✓		✓
	MCX1 (rep)											

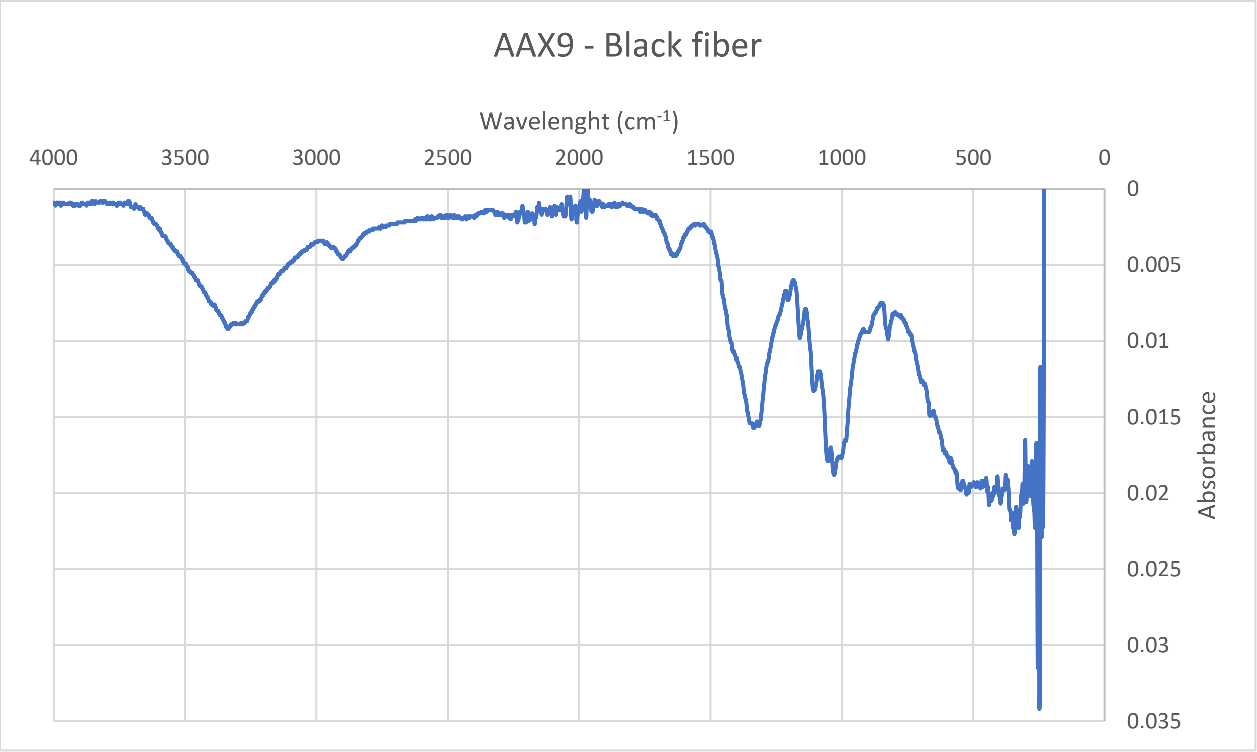

Figure S1. Example of FTIR spectra of a fibre of the GIT filter.

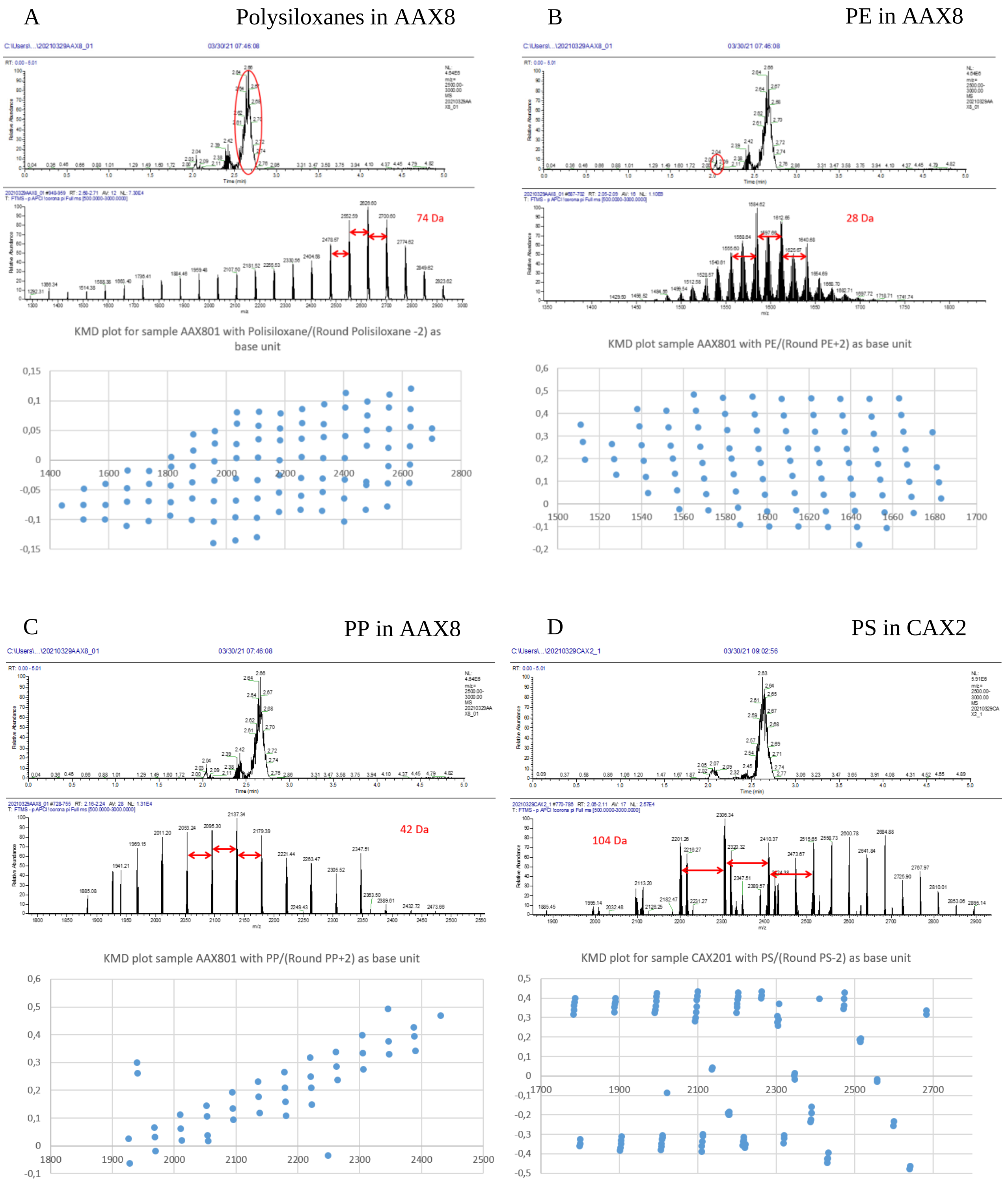

Figure S2. Examples of chromatograms, mass spectra and KMD plots of identified polymers. Polysiloxanes (A), PE (B), PP (C), and PS (D).

1. Setala, O., V. Fleming-Lehtinen, and M. Lehtiniemi, *Ingestion and transfer of microplastics in the planktonic food web.* Environ Pollut, 2014. **185**: p. 77-83.
2. Ormsby, R.T., et al., *Evidence that osteocyte perilacunar remodelling contributes to polyethylene wear particle induced osteolysis.* Acta Biomaterialia, 2016. **33**: p. 242-251.
